# Role of Polyamine-Induced Dimerization of Antizyme in Its Cellular Functions

**DOI:** 10.3390/ijms23094614

**Published:** 2022-04-21

**Authors:** Mervi T. Hyvönen, Olga A. Smirnova, Vladimir A. Mitkevich, Vera L. Tunitskaya, Maxim Khomutov, Dmitry S. Karpov, Sergey P. Korolev, Merja R. Häkkinen, Marko Pietilä, Marina B. Gottikh, Jouko Vepsäläinen, Leena Alhonen, Alexander A. Makarov, Sergey N. Kochetkov, Heather M. Wallace, Tuomo A. Keinänen, Alex R. Khomutov

**Affiliations:** 1 School of Pharmacy, Biocenter Kuopio, University of Eastern Finland, Yliopistonranta 1B, 70210 Kuopio, Finland; merja.hakkinen@thl.fi (M.R.H.); jouko.vepsalainen@uef.fi (J.V.); alhonenl@gmail.com (L.A.); tuomo.keinanen@uef.fi (T.A.K.); 2 Engelhardt Institute of Molecular Biology, Russian Academy of Sciences, Vavilov Street 32, 119991 Moscow, Russia; o.smirnova.imb@gmail.com (O.A.S.); mitkevich@gmail.com (V.A.M.); ve_tun@mail.ru (V.L.T.); makhomutov@mail.ru (M.K.); aleom@yandex.ru (D.S.K.); aamakarov@eimb.ru (A.A.M.); snk1952@gmail.com (S.N.K.); 3 Belozersky Institute of Physico-Chemical Biology, Lomonosov Moscow State University, Leninskie Gory 1, 119991 Moscow, Russia; spkorolev@mail.ru (S.P.K.); mgottikh@gmail.com (M.B.G.); 4 School of Medicine, Biocenter Kuopio, University of Eastern Finland, Yliopistonranta 1E, 70210 Kuopio, Finland; marko.pietila@uef.fi; 5 School of Medicine, Medical Sciences and Nutrition, Institute of Medical Sciences, University of Aberdeen, Foresterhill, Aberdeen AB25 2ZD, UK; h.m.wallace@abdn.ac.uk

**Keywords:** polyamines, antizyme, dimerization, polyamine analogues, ribosomal frameshifting, polyamine uptake, ornithine decarboxylase, α-difluoromethylornithine

## Abstract

The polyamines, spermine (Spm) and spermidine (Spd), are important for cell growth and function. Their homeostasis is strictly controlled, and a key downregulator of the polyamine pool is the polyamine-inducible protein, antizyme 1 (OAZ1). OAZ1 inhibits polyamine uptake and targets ornithine decarboxylase (ODC), the rate-limiting enzyme of polyamine biosynthesis, for proteasomal degradation. Here we report, for the first time, that polyamines induce dimerization of mouse recombinant full-length OAZ1, forming an (OAZ1)_2_-Polyamine complex. Dimerization could be modulated by functionally active *C*-methylated spermidine mimetics (MeSpds) by changing the position of the methyl group along the Spd backbone—2-MeSpd was a poor inducer as opposed to 1-MeSpd, 3-MeSpd, and Spd, which were good inducers. Importantly, the ability of compounds to inhibit polyamine uptake correlated with the efficiency of the (OAZ1)_2_-Polyamine complex formation. Thus, the (OAZ1)_2_-Polyamine complex may be needed to inhibit polyamine uptake. The efficiency of polyamine-induced ribosomal +1 frameshifting of OAZ1 mRNA could also be differentially modulated by MeSpds—2-MeSpd was a poor inducer of OAZ1 biosynthesis and hence a poor downregulator of ODC activity unlike the other MeSpds. These findings offer new insight into the OAZ1-mediated regulation of polyamine homeostasis and provide the chemical tools to study it.

## 1. Introduction

The polyamines, spermidine (Spd) and spermine (Spm), are essential, ubiquitous, and organic polycations present in all eukaryotic cells in μM–mM concentrations and are involved in the regulation of numerous vital processes required for the differentiation, proliferation, and other cellular functions [1]. Disturbance of polyamine metabolism is associated with many diseases, including malignant tumors (cancer cells have elevated level of polyamines), decreased immune response, some types of pancreatitis, Snyder–Robinson’s syndrome, and Alzheimer’s and Parkinson’s disease [1,2,3,4,5,6,7,8]. Polyamine levels are precisely regulated through a series of feedback circuits to maintain cellular functions without leading to a malignant transformation of the cell. Ornithine decarboxylase (ODC), the rate-limiting enzyme of polyamine biosynthesis is one of the most short-lived proteins known, having a half-life of 10–20 min. Its activity and half-life are regulated by a family of antizyme proteins of which antizyme 1 (OAZ1) is the most abundant [9]. ODC is active only as a homodimer, and the dissociation by OAZ1 leads to a loss of the activity. OAZ1 binds to one of the ODC subunits and targets it for ubiquitin-independent degradation by the 26S proteasome [10]. In addition, OAZ1 regulates both the uptake and export of polyamines [11]. Apart from the regulation of polyamine homeostasis, ОAZ1 has been reported to accelerate the ubiquitin-independent degradation of important regulatory proteins, such as Aurora-A kinase [12], Smad1 [13], and Cyclin D1 [14,15], although for the last one contradictory data also exist [16]. OAZ1 is also known to increase the activity of ATP citrate lyase through enzyme–OAZ1 interaction that makes OAZ1 a “sort of an additional bridge” between polyamine and acetyl-CoA metabolism [17].

The biosynthesis of OAZ1 is stimulated by polyamines via the promotion of +1 ribosomal frameshift on OAZ1 mRNA, which is needed for the read-through of a stop codon [18,19]. The nucleotide sequence around the frameshifting site is well-conserved among eukaryotes [20]. Three *cis*-acting elements are known to stimulate mammalian OAZ1 frameshifting: the stop codon, a 50-nucleotide sequence immediately before the shift site, and a pseudoknot starting three nucleotides after the stop codon [18,21]. However, it is not completely understood how the frameshifting event is regulated by polyamines.

Here we found, surprisingly, that Spm and Spd induce dimerization of full-length OAZ1 protein with the formation of the (OAZ1)_2_-polyamine complex. Dimerization could be modulated by moving the methyl group along the backbone of functionally active *C*-methylated Spd mimetics—2-MeSpd was a poor inducer as opposed to 1-MeSpd, 3-MeSpd, and Spd, which were good inducers. Moreover, we demonstrate that the efficiency of the (OAZ1)_2_-Polyamine complex formation correlates well with the ability of the compound to inhibit polyamine uptake. Finally, functionally active *C*-methylated Spd mimetics differently induced ribosomal +1 frameshifting of OAZ1 mRNA and hence the production of full-length OAZ1. The induction of OAZ1 biosynthesis correlated with the compound’s ability to downregulate ODC activity. These findings reveal novel interconnecting molecular features in the OAZ1-dependent maintenance of polyamine homeostasis and provide the chemical tools with which to study it.

## 2. Results

### 2.1. Polyamines Induce mOAZ1 Dimerization In Vitro

We first investigated the interaction of natural polyamines and the analogue, DENSpm (*N*^1^,*N*^11^-diethylnorspermine) with mouse OAZ1 (mOAZ1) using isothermal titration calorimetry (ITC) (Table 1). Full-length mOAZ1 protein was successfully produced in *E. coli* (Figures S1 and S2). Representative ITC titration curves and binding isotherms for polyamines interacting with mOAZ1 are shown in Figure S3. We found that Spd, Spm, and DENSpm bound to mOAZ1, whereas under the conditions used, putrescine (Put) did not. Surprisingly, the stoichiometry of polyamine binding to mOAZ1 was 1:2, i.e., the dimeric (mOAZ1)_2_-Polyamine complex was formed (Table 1). The association constants (*K*_a_) for the complexes of Spm and DENSpm with mOAZ1 were about threefold lower than that for Spd. The thermodynamic parameters of complex formation (Δ*H* and *T*Δ*S* ratio) were virtually similar for all three polyamines, indicating the same character of their binding with mOAZ1.

**Figure 1:**
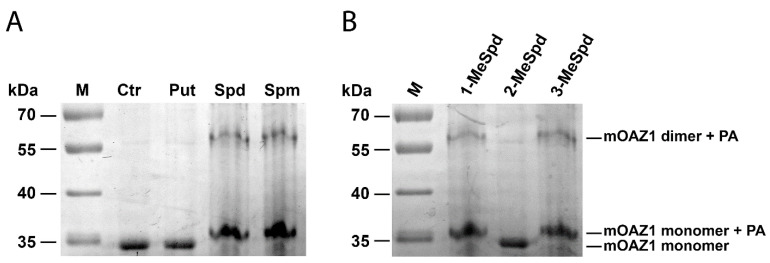
Formation of (mOAZ1)_2_-Polyamine (PA) complex. (**A**) Spd and Spm induce dimerization of OAZ1. The recombinant mOAZ1 protein (1 µg/band) was incubated in the presence of 200 μM of polyamines for 30 min at 20 °C at pH 7.5, and the reaction mixtures were resolved on a 12% polyacrylamide gel (without SDS) in the presence of 0.01% SDS in the running buffer with a subsequent staining with Coomassie Blue R-250. (**B**) 1-MeSpd and 3-MeSpd, but not 2-MeSpd, bind to mOAZ1 and induce its dimerization. The conditions of the complex formation and electrophoresis are the same as in Figure 1A.

The existence of the (mOAZ1)_2_-Polyamine complex was also confirmed by Laemmli electrophoresis without SDS in the separating gel and having 0.01% SDS in the running buffer that allowed to detect the band corresponding to this complex (Figure 1A). In addition to the dimeric complex, a lot of the monomeric OAZ1-Polyamine complex was detected, most probably because the (mOAZ1)_2_-Polyamine complex may partly dissociate in the presence of 0.01% SDS in the running buffer. Taken together, these results indicate that polyamines have a high affinity to the full-length mOAZ1 and form the (mOAZ1)_2_-Polyamine complex.

### 2.2. Modulation of (mOAZ1)_2_-Polyamine Complex Formation with MeSpds

Most of Spd and Spm analogues, and even those of an oligoamine nature, induce OAZ1 synthesis in cells [22,23]. *C*-Methylated analogues of Spd and Spm are functionally active mimetics capable of fulfilling many physiological functions of the natural polyamines [24]. The interaction of these analogues with the enzymes of polyamine metabolism and their cellular effects may be modulated by moving of the methyl substituent along the polyamine backbone [25,26]. Therefore, having discovered the OAZ1 dimerization phenomenon, we attempted to modulate the formation of the (OAZ1)_2_-Polyamine complex using a set of functionally active mimetics of Spd, namely, 1-MeSpd, 2-MeSpd, and 3-MeSpd.

The ITC data clearly showed that the position of the methyl group in the Spd analogue crucially influenced the ability to bind to and induce the dimerization of mOAZ1 (Table 1, Figure S3). The interaction of mOAZ1 with 1-MeSpd or with 3-MeSpd was characterized by the *K*_d_ values, which were close to that observed for Spd, i.e., about 10^−6^ M. However, the binding of 2-MeSpd to mOAZ1 was not detected. The existence of (mOAZ1)_2_‒MeSpd complexes in the case of 1-MeSpd and 3-MeSpd were also investigated as above by electrophoresis using 0.01% SDS in the running buffer. As shown in Figure 1B, dimeric and monomeric complexes of mOAZ1 with 1-MeSpd and 3-MeSpd were detected. However, in the case of 2-MeSpd, not even the monomeric mOAZ1‒2-MeSpd complex was detected under these conditions, and only the band corresponding to mOAZ1 itself was observed (Figure 1B). Therefore, the formation of an (OAZ1)_2_-Polyamine complex can be regulated by moving the methyl group along the backbone of functionally active *C*-methylated Spd mimetics.

### 2.3. MeSpds Differently Induce +1 Frameshifting of OAZ1 mRNA

**Figure 2:**
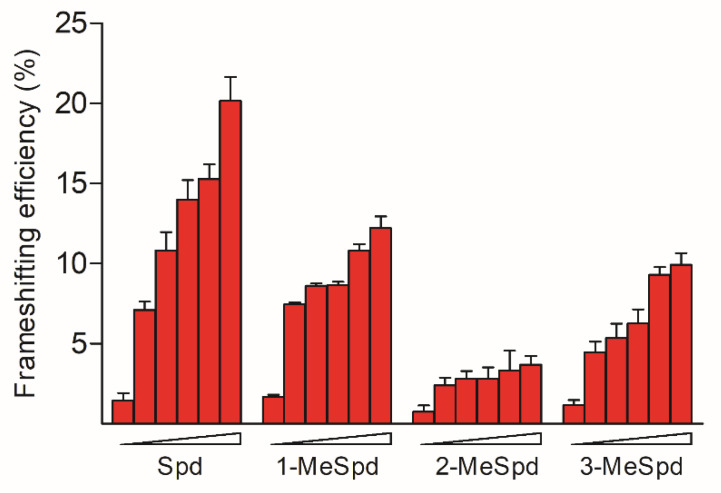
The effects of Spd and its *C*-methylated derivatives on the efficiency of the +1 frameshifting of eukaryotic OAZ1 mRNA. Average percent frameshifting in the *spe*1, *spe*2, *paa*1, and *fms*1 deletant strain of *S. cerevisiae* IS532/44d was measured using a bicistronic assay and plotted versus polyamine concentrations (0, 0.25, 0.5, 1, 2, and 4 mM) in the medium. The IS532/44d strain of *S. cerevisiae* was transformed with pAC98T-OAZ1-FS or pAC98T-OAZ1-Cont plasmids, and luciferase activity was measured and then normalized to β-galactosidase activity essentially as described in [28]. Error bars indicate standard deviations for three independent transformants, analyzed in triplicate.

It is well known that polyamines induce the +1 frameshift of OAZ1 mRNA required for the synthesis of full-length protein [27]. Thus, we next investigated the ability of Spd and its *C*-methylated analogues to promote +1 ribosomal frameshift using the earlier developed bicistronic frameshift reporter system [28]. The system consists of the quadruple *S. cerevisiae* mutant strain IS532/44d, which is unable to synthesize and degrade polyamines, and the plasmid containing the reporter β-galactosidase gene and the firefly luciferase gene fused with a fragment of the eukaryotic OAZ1 frameshift site. The frameshifting activity of 1-MeSpd at 0.25 mM was about the same as Spd, while at 2.0 mM and 4.0 mM both 1-MeSpd and 3-MeSpd were weaker inducers of the frameshifting than Spd (Figure 2). Importantly, 2-MeSpd was a substantially weaker inducer of +1 frameshift event than the other compounds at all concentrations tested.

### 2.4. MeSpds Differently Stabilize the Stem–Loop Region of mOAZ1 mRNA

It is suggested that polyamines can stabilize the hairpin structure of the OAZ1 mRNA, located just after the +1-frameshifting site and thereby induce a +1 frameshift [27]. Here we investigated the effects of Spd and its *C*-methylated analogues on the melting temperature of model 72-mer-2′-O-Me-oligoribonucleotide containing a +1-frameshift site, hairpin, and pseudoknot of the mOAZ1 mRNA. Spd and its mimetics induced stabilization of the oligonucleotide in a concentration-dependent manner, as evidenced by the increased melting temperature as the analogue concentration increased from 20 μM up to 200 μM (Table S1). The most noticeable differences in the effect of the tested *C*-methylated Spd analogues on the stability of the oligonucleotide were observed at the Spd–analogue concentration of 200 µM. 2-MeSpd stabilized the hairpin structure of this region of OAZ1 mRNA less if compared with that of Spd and the other analogues—the observed *T*_m_ values being 2–3 °C lower for 2-MeSpd (Table S1).

### 2.5. MeSpds Differently Affect OAZ1 Synthesis and ODC Activity in DU145 Cells

**Figure 3:**
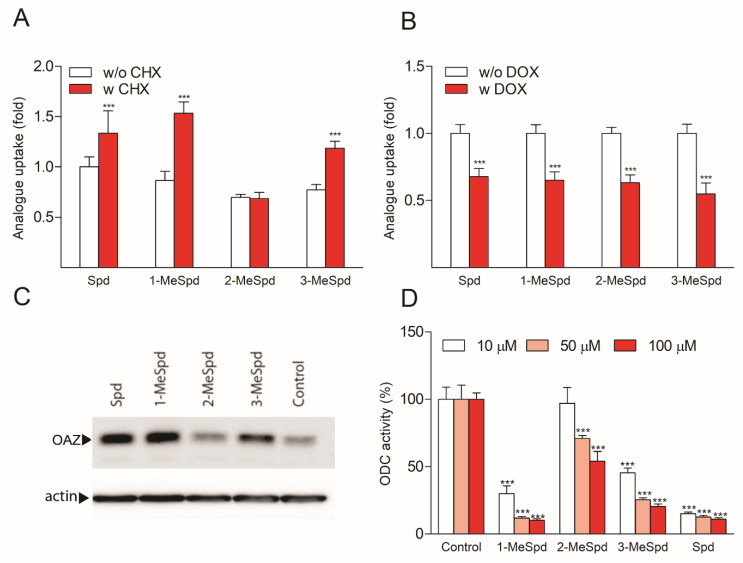
2-MeSpd is the weakest inducer of OAZ1 synthesis and the weakest downregulator of ODC in DU145 cells. (**A**) Uptake of Spd and its analogues in CHX-treated DU145 cells. After 2 h preincubation with CHX (100 µg/mL), the compounds (100 µM) were added, and incubation continued for 4 h before the analysis of the intracellular analogue concentration. (**B**) Uptake of Spd and its analogues in OAZ1-overexpressing DU145 cells. OAZ1 was induced by 24 h preincubation with DOX (250 ng/mL). The compounds (100 µM) were added for further 6 h before the analysis of intracellular analogue concentration. (**C**) Effect of Spd and the analogues on OAZ1 protein levels in DU145 cells. The cells were incubated for 4 h with 100 μM analogues in the presence of the proteasomal inhibitor, MG132 (25 μM). (**D**) Effect of Spd and the analogues on ODC activity in DU145 cells. The cells were incubated with the analogues or Spd for 6 h, after which the cells were harvested for measurements of ODC activity. All plates (**A**–**D**) were supplemented with 1 mM aminoguanidine (AG, an inhibitor of serum amino oxidases) to prevent the degradation of Spd to toxic metabolites. Results are means ± SD, *n* = 3 biologically independent samples, representative of *n* = 3 independent experiments. *** refers to the statistical significance of *p* < 0.001.

To investigate the effects of Spd and its *C*-methylated analogues on the induction of OAZ1 biosynthesis, we used two methods. In the first, indirect method, DU145 prostate cancer cells were incubated in the presence or the absence of cycloheximide (CHX), which nonspecifically inhibits protein biosynthesis, including that of the very short-lived OAZ1. All the tested compounds, except 2-MeSpd, accumulated in higher amounts in CHX-treated compared to untreated cells (Figure 3A), suggesting that 2-MeSpd may be a poor inducer of OAZ1 biosynthesis. The possibility that OAZ1 was induced by 2-MeSpd, but OAZ1 was somehow unable to limit the uptake of 2-MeSpd was ruled out by using OAZ1-overexpressing DU145 cells. Doxycycline (DOX)-induced expression of OAZ1 reduced the uptake of all compounds tested, including that of 2-MeSpd (Figure 3B). We also analyzed the effect of analogues on the biosynthesis of the OAZ1 protein in DU145 cells pretreated with the proteasomal inhibitor MG132. Western blotting analysis indicated that the OAZ1 protein level was only marginally induced upon 4 h incubation of the cells with 2-MeSpd, whereas the other *C*-methylated Spd analogues and Spd showed a clear induction (Figure 3C).

Since OAZ1 is the key regulator of ODC stability and activity, we also investigated the effect of Spd and its analogues on ODC activity in DU145 cells. As depicted in Figure 3D, ODC activity was downregulated in a dose-dependent manner by all analogues tested, but 2-MeSpd was about a 10-fold less efficient downregulator than 1-MeSpd, 3-MeSpd, and Spd. One may argue that the observed differences in the ability of *C*-methylated Spd analogues to induce OAZ1 biosynthesis and to downregulate ODC were attributed to the differences in the intracellular accumulation of the analogues. However, an analysis of the intracellular polyamine pools demonstrated that the cells treated with 3-MeSpd had the lowest accumulation of the analogue and displayed the lowest total polyamine concentration (Spd + Spm + analogue) (Table S2), despite showing efficient induction of OAZ1 and downregulation of ODC. These results indicate that neither the concentration of the intracellular analogue nor the total polyamine concentration could explain the observed difference in the OAZ1 induction efficacy between 2-MeSpd and other *C*-methylated Spd analogues. Thus, the ability of the compounds to induce OAZ1 biosynthesis correlated with their ability to downregulate ODC activity.

The direct addition of Spd or the analogues (1 mM) to the ODC assay mixture containing ODC-enriched cell sample (subconfluent DU145 cells collected 6 h after medium change) did not affect ODC activity in vitro (Figure S4A), indicating that polyamines do not directly inhibit the enzyme. Moreover, when ODC-enriched cytosolic extract was incubated with cytosolic extract from OAZ1-overexpressing cells, the supplementation of either Spd or its *C*-methylated analogues did not affect the ability of OAZ1 to inhibit ODC activity in vitro (Figure S4B).

### 2.6. (OAZ1)_2_-Polyamine Complex Downregulates Polyamine Uptake in DU145 Cells

**Figure 4:**
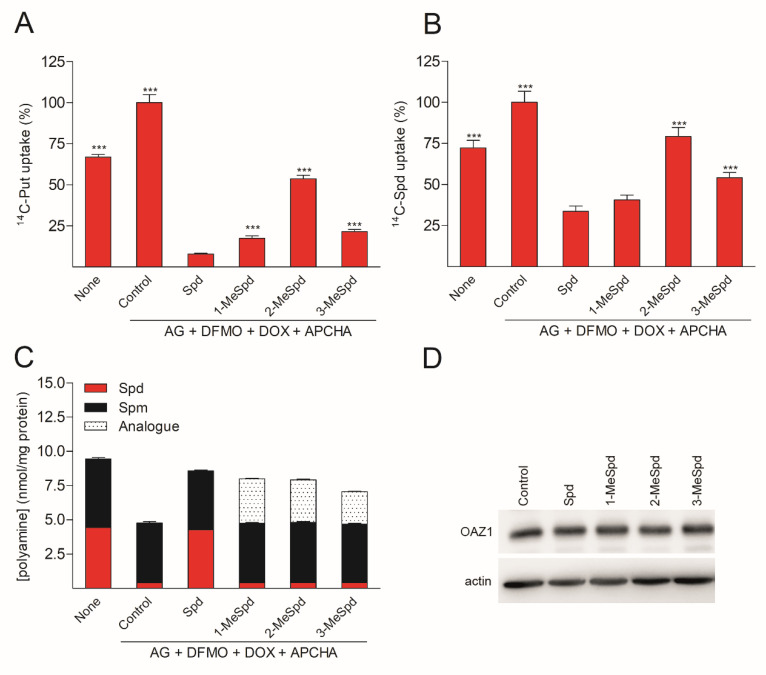
2-MeSpd is the weakest downregulator of Put and Spm uptake in DU145 cells. (**A**) [^14^C]-Put and (**B**) [^14^C]-Spd uptake in OAZ1-overexpressing DU145 cells. The cells were first treated with DFMO (5 mM) for 3 days, after which DOX (1 μg/mL) was added for further 2 days to induce OAZ1 overexpression. Then, AG (1 mM), APCHA (50 μM), and Spd/analogue (100 μM) were added for 4 h. The plates were washed, and 20 min [^14^C]-Put or [^14^C]-Spd (10 μM) uptake assays were performed. ***, *p* < 0.001 as compared to Spd-treated group. (**C**) Intracellular polyamine and analogue pools before [^14^C]-Put/Spd uptake assays. (**D**) OAZ1 protein amount before [^14^C]-Put/Spd uptake assays. Results are means ± SD, *n* = 3 biologically independent samples, representative of *n* = 3 independent experiments.

To investigate whether the formation of the (OAZ1)_2_-Polyamine complex plays a role in the regulation of polyamine uptake, we used OAZ1-overexpressing cells (containing a mutation in the stop codon, so that the cells produce OAZ1 without the need for frameshifting) to exclude the effect of the analogues on OAZ1 biosynthesis. The cells were first pretreated with α-difluoromethylornithine (DFMO, an irreversible inhibitor of ODC) for 3 days in order to deplete the Spd pool, and the incubation with Spd or the analogues was carried out in the presence of *N*-(3-aminopropyl)cyclohexylamine (APCHA), an inhibitor of spermine synthase, in order to prevent the conversion of MeSpd to MeSpm. Before the uptake assay, the plates were washed free of compounds. As depicted in Figure 4, treatment with 2-MeSpd clearly induced the weakest downregulation of both [^14^C]-Put and [^14^C]-Spd uptake. Importantly, the difference between the compounds in their ability to induce downregulation of [^14^C]-Put/Spd uptake was not related to the total polyamine levels, the amount of the analogue (Figure 4C), or the amount of OAZ1 (Figure 4D). Therefore, the formation of the (OAZ1)_2_-Polyamine complex may be required for the inhibition of polyamine uptake, which is an essential cellular function of OAZ1.

### 2.7. Polyamines Do Not Stabilize OAZ1 Protein in DU145 Cells

Finally, we investigated whether Spd or its *C*-methylated analogues could stabilize mammalian OAZ1 protein, as was reported for yeast OAZ1 with Spd [29]. We used OAZ1-overexpressing DU145 cells to exclude the effect of analogues on OAZ1 biosynthesis. As depicted in Figure S5, none of the tested compounds markedly influenced OAZ1 degradation, at least under the experimental conditions used, and no correlation between the formation of the (OAZ1)_2_-Polyamine complex and the stability of OAZ1 was observed in DU145 cells.

## 3. Discussion

A high intracellular concentration of polyamines (up to low mM) a priori determines the diversity of their cellular functions [30]. Intracellular polyamine content is strictly controlled through differential feedback mechanisms in both positive and negative directions. A short-lived protein, OAZ1, is a key downregulator of polyamine pools. This protein has a high affinity toward the ODC subunit, and the resulting interaction inhibits ODC dimerization and thus activity. In addition, OAZ1 induces a conformational change in ODC leading to the exposure of the unstructured *C*-terminus of ODC that is then recognized by 26S proteasome [31]. Normally the intracellular amount of OAZ1 is very low, less than 1 ng/mg of total cytosolic protein in rat liver [32]. Polyamines noticeably increase the +1-frameshifting efficiency needed to read through the stop codon of OAZ1 mRNA, allowing the synthesis of the full-length protein. Hence, OAZ1 is synthesized and functions in cells with elevated polyamine concentrations.

Most cellular polyamines are bound to nucleic acids, mainly to RNA and nucleoprotein complexes [33]. However, specific interactions of Spm and Spd with different proteins are also of crucial importance for cell viability and metabolism [34]. Polyamines are essential for polymerization of α/β-tubulin heterodimers, having negatively charged *C*-terminal ends, into microtubules. The depletion of the cellular polyamine pool leads to decreased microtubule mass, while the addition of Spm reverses this effect [35]. Another example is α-synuclein, a small 140 aa protein, which is abundantly expressed in the central nervous system and involved in the regulation of lipid metabolism and dopamine synthesis and transport. Native α-synuclein is soluble and unfolded, but its fibrillar aggregates are a hallmark in Parkinson’s disease (PD). A link between the deregulation of Spm metabolism and PD histopathology was confirmed using a mouse model overexpressing α-synuclein: the depletion of Spm alleviated PD histopathology by reducing α-synuclein aggregation, whereas the excess of Spm increased aggregate formation [2]. One more example of polyamine–protein interaction is the polyamine-promoted autocatalysis of nucleolin [36]. Nucleolin is an RNA-binding protein, which stabilizes the mRNA stem–loop in the initiation region of spermidine/spermine-*N*^1^-acetyltransferase (SSAT), the rate-limiting enzyme of Spm and Spd catabolism. Nucleolin binding represses SSAT translation, whereas polyamines promote nucleolin autocatalysis, thereby relieving nucleolin repression.

Previous studies have utilized *N*-terminally truncated eukaryotic OAZ1s, which are more stable, soluble, and retain the affinity to the ODC subunit. The structure of rat OAZ1^(87−227)^ was studied by NMR [37], and the structure of the complex of the ODC with human OAZ1^(95−228)^ was studied by X-ray [38]. However, it is not known whether the *N*-terminal region of OAZ1 plays a role in polyamine sensing. Most of the studies of OAZ1 protein have been performed in the absence of polyamines, although OAZ1 is synthesized and functions in cells with elevated polyamine concentrations. The only exception is an earlier study utilizing an ultrafiltration-based system with radiolabeled polyamines, which showed that Spd binds to yeast OAZ and to the maltose-binding protein-fused human OAZ1 [39]. However, the stoichiometry and thermodynamic parameters of the binding were not examined.

In the present study, we successfully produced full-length mOAZ1 protein to study its interaction with polyamines. We found that polyamines have a high affinity to the full-length mOAZ1 and that they induce the formation of the (mOAZ1)_2_-Polyamine complex with a *K*_d_ value of ~10^−6^ M (Table 1, Figure 1A). These key experiments were performed in the presence of 150 mM KCl, but in the absence of Mg^2+^, the most abundant divalent ion in mammalian cells, having a free intracellular concentration higher than that of any other ion (0.5–1 mM) [40]. By interacting with RNA, polyamines stimulate protein synthesis and decrease the optimal Mg^2+^ concentration, indicating that polyamines and Mg^2+^ can partially substitute for each other in protein synthesis. However, polyamines fulfil many different and vitally important regulatory functions in cells, including covalent modifications of proteins [34], and they are not directly analogous to divalent inorganic cations, as their interactions also include hydrogen bonding and hydrophobic forces in a flexible alkyl chain. It is known that some proteins such as calmodulin, troponin C, parvalbumin, and S100 protein can bind substantial amounts of Mg^2+^ [40], but in general Mg^2+^ binds weakly to proteins and enzymes (*K*_a_ ≤ 10^−5^ M) [41]. To our best knowledge, there are no data available about the binding of Mg^2+^ to mammalian OAZ1. Thus, in vitro studies examining the effect of Mg^2+^ on the interaction of polyamines with OAZ1 are warranted. The ability of polyamines to dimerize mOAZ1 was completely unexpected, as OAZ1 has been earlier considered to exist only in the monomeric form. The high intracellular concentration of polyamines in the living cells suggests that (mOAZ1)_2_-Polyamine complexes should exist in vivo.

The above results prompted us to study the OAZ1-Polyamine interaction further using *C*-methylated analogues of Spd to dissect the structure–activity determinants and possible novel physiological functions of polyamine binding to OAZ1. *C*-methylated analogues of Spd belong to a family of functionally active, noncytotoxic mimetics of Spd, which are useful tools to investigate the metabolism and functions of polyamines. Their interaction with the enzymes of polyamine metabolism may be regulated by moving the methyl substituent along the Spd backbone. For example, 1-MeSpd is not a substrate of SSAT, but is a substrate of spermine synthase (SpmSy) [25]. On the contrary, 3-MeSpd is not a substrate of either SSAT or SpmSy, i.e., it is metabolically stable, while 2-MeSpd is a substrate of both SSAT and SpmSy [25]. The effects of the mimetics on cell growth and on the hypusination status of eIF5A during prolonged polyamine deprivation [42] and on the adipogenic differentiation of 3T3-L1 cells [43,44] differ depending on the position of the methyl group in the analogue. The distinct ability of MeSpds to functionally replace Spd in the biofilm formation of *Bacillus subtilis* was also shown [45]. Furthermore, 1-MeSpd has been tested as a therapeutic in mice and rats in various disease models, such as acute pancreatitis, endotoxemia, and liver damage. Due to its catabolic stability and ability to function as Spd mimetic, the analogue prevented acute pancreatitis and restored liver regeneration in transgenic rats with activated polyamine catabolism [46,47,48]. 1-MeSpd was also shown to activate the hair follicle cycle in mice [49], and it has been tested in clinical trials against hair loss [50].

Here, the experiments surprisingly show that among the tested MeSpds, 2-MeSpd was unable to dimerize mOAZ1 (Table 1, Figure 1B). This means that the efficiency of the (OAZ1)_2_–MeSpd complex formation can be modulated by moving the methyl group along the Spd backbone. This opened the possibility to use these analogues as a tool to investigate the role of OAZ1 dimerization in its cellular functions, i.e., the downregulation of ODC activity and inhibition of polyamine uptake. When the Spd pool of OAZ1 overexpressing DU145 cells was replaced with *C*-methylated Spd analogue, 2-MeSpd was the weakest inducer of the inhibition of polyamine uptake (Figure 4). This important finding indicated that not only the amount of newly synthesized OAZ1, but also the formation of the (OAZ1)_2_-Polyamine complex may be required for the inhibition of polyamine uptake.

We also considered the possibility that the formation of the (OAZ1)_2_-Polyamine complex might increase the half-life of the OAZ1 protein, since polyamines have been shown to stabilize *S. cerevisiae* OAZ1 against proteasomal degradation [29,51]. However, none of the Spd analogues affected OAZ1 protein stability in the DU145 cells, at least under the experimental conditions used. Our results are in agreement with earlier findings in HTC cells, where Spm and SL-11144 oligoamine did not increase the half-life of OAZ1 protein [52].

In experiments concerning another key cellular function of OAZ1, the downregulation of ODC activity, we found that 2-MeSpd was more than 10-fold less effective at downregulating ODC activity than Spd, 1-MeSpd, and 3-MeSpd (Figure 3D). Using Western blot analysis, we confirmed that the reason for the weak ODC downregulation was a poor induction of OAZ1 synthesis by 2-MeSpd (Figure 3C).

Utilizing a dual frameshifting reporter system containing eukaryotic OAZ1 frameshift site, we found that 2-MeSpd was a much weaker inducer of OAZ1 synthesis compared to Spd, 1-MeSpd, or 3-MeSpd (Figure 2). The exact molecular mechanism of polyamine-induced +1 frameshifting of OAZ1 mRNA is still unknown, although the effect was discovered already in the 1990s [27]. Polyamines may stabilize the stem–loop structure of mRNA starting after the stop codon [53] and/or interact with 5′-element [21] in such a way that these interactions slow down the elongation speed of the ribosome allowing it to pass through the stop codon. In the case of *S. cerevisiae,* it was demonstrated that polyamines interact with the nascent chain of OAZ1, affecting the downstream frameshifting event [39]. However, mouse and yeast OAZ1 proteins show less than 10% sequence identity and differ in size (25 kDa and 34 kDa). Therefore, these data cannot be directly translated to highly conserved mammalian OAZ1. However, the interaction of polyamines with the OAZ1 growing chain might be related to the dimerization of OAZ1 and might be of interest when interpreting the weak potency of 2-MeSpd in inducing +1 frameshifting of OAZ1 mRNA. Poor stabilization of the stem–loop structure of mRNA starting after the stop codon by 2-MeSpd may be another reason why this analogue was a poor inducer of OAZ1 synthesis. According to our RNA melting temperature analysis, 2-MeSpd was a weaker stabilizer of the hairpin structure of model 72-mer 2′-O-Me-oligoribonucleotide of the mOAZ1 mRNA (Table S2). This might be an indication of the importance of the second position of the Spd chain for the interaction with OAZ1.

Taken together, here we demonstrate for the first time that the interaction of full-length OAZ1 protein with polyamines results in the formation of the (OAZ1)_2_-Polyamine complex, which correlates well with cellular functions of OAZ1, i.e., inhibition of polyamine uptake. In addition, we demonstrate that the efficiency of +1 frameshift of OAZ1 mRNA, which is required for the synthesis of full-length protein, can be modulated by moving the methyl group along the backbone of functionally active *C*-methylated mimetics of Spd. The findings obtained offer a new insight into the OAZ1-mediated regulation of polyamine homeostasis and provide chemical tools with which to study it.

## 4. Materials and Methods

### 4.1. Materials

1-MeSpd was synthesized according to a previously published method [54], 2-MeSpd and 3-MeSpd as described in [25]. DENSpm was synthesized essentially as described in [55], and APCHA was synthesized as in [56]. DFMO was a kind gift from Dr. Patrick Woster (Medical University of South Carolina, Charleston, SC, USA). The sequence of mouse OAZ1 was from [57]. Model 72-chain-2′-O-Me-oligonucleotide mOAZ1-PK (5′-UGG UGC UCC UGA UGU CCC UCA CCC ACC CCU GAA GAU CCC AGG UGG GCG AGG GAA CAG UCA GCG GGA UCA CAG-3′) was purchased from DNA-Synthesis LLC (Moscow, Russia). Plasmids pAC98T-OAZ1-FS and pAC98T-OAZ1-Cont and quadruple *Saccharomyces cerevisiae* mutant were a kind gift from Prof. Ian Stansfield (University of Aberdeen, Scotland, UK).

### 4.2. Plasmid Construction

The plasmid pET-21-2c-mAZ for expression of mouse antizyme with *C*-terminal His-tag (Figure S1) was constructed by amplification of OAZ1-encoding region from the plasmid pQE30-mAZ [57] (a kind gift of Prof. O. Jänne, University of Helsinki, Finland) using oligonucleotides (5′-TATGAATTCAATGGTGAAATCCTCCCTGC-3′) and oligonucleotide (5′-TATCTCGAGGTCCTCCTCACCCGGGT-3′), digestion of the product with *EcoR*I and *Xho*I endonucleases, and cloning into the respective sites of a two-cistron pET-21-2c vector described previously [58]. Structure of constructed plasmid was confirmed by sequencing.

### 4.3. The Expression and Purification of mOAZ1 Protein

Rosetta (DE3) *Escherichia coli* strain (Novagen, St. Louis, MO, USA) was transformed with the plasmid pET-21-2c-mAZ encoding *C*-terminally His-tagged protein. The cells bearing the target plasmid were grown in 5 mL of Lisogeny broth (LB) medium supplemented with 150 mg/L ampicillin (A150) and 15 mg/L chloramphenicol (C15) at 37 °C overnight. An aliquot of 2 mL was harvested by centrifugation (3200× *g*, 10 min); the pellet was resuspended in 500 mL of fresh LB medium supplemented with A150 and C15, and the cells were grown at 37 °C. When optical density at 550 nm reached 0.5, isopropyl-β-D-thiogalactopyranoside was added to a final concentration of 1 mM, and the cells were grown for additional 3 h and harvested by centrifugation (3200× *g*, 15 min) at 4 °C. The cell pellet was resuspended on ice in 20 mL of buffer A (25 mM Tris–HCl, pH 7.5, 350 mM NaCl, 10% (*v*/*v*) glycerol, and 1 mM 2-mercaptoethanol) and supplemented with 0.5% (*v*/*v*) Triton X-100, protease inhibitors phenylmethylsulfonyl fluoride (PMSF, 1 mM), and a Protease inhibitor cocktail (P8849, Sigma, St. Louis, MO, USA). The lysate was sonicated on ice by ten 46 s impulses with 90 s intervals. After removal of cell debris by centrifugation (8200× *g*, 15 min) at 4 °C, the clarified lysate was applied onto a 2 mL column with Ni-NTA-agarose (Novagen) pre-equilibrated with the buffer A. The resin was washed with buffer A supplemented with the protease inhibitors and increasing concentrations of imidazole (10, 30, and 50 mM) (15 mL each), and the protein was eluted with the same buffer with 200 mM imidazole; 1 mL fractions were collected, and the protein was assessed using Bradford reagent. The fractions containing highest levels of the protein were pooled and dialyzed against buffer B (25 mM potassium phosphate, pH 7.5, 300 mM KCl, 5% (*v*/*v*) glycerol, 1 mM 2-mercaptoethanol, and 1 mM PMSF) for 4 h and then against buffer C (25 mM potassium phosphate, pH 7.5, 150 mM KCl, 50% (*v*/*v*) glycerol, 1 mM 2-mercaptoethanol, and 1 mM PMSF) overnight. The yield of *C*-terminally-tagged antizyme was 7 mg per 1 L of the culture.

The purified mOAZ1-6xHis eluted from Ni-NTA-agarose column was practically homogeneous as shown by SDS-PAGE electrophoresis (Figure S2).

### 4.4. Isothermal Titration Calorimetry (ITC)

For the precise isothermal titration calorimetry (ITC) experiments only freshly purified samples turned out to be suitable. The thermodynamic parameters of polyamines binding to OAZ1 were measured using a MicroCal iTC200 instrument (GE Healthcare, Chicago, IL, USA) as described elsewhere [59]. Experiments were carried out at 31 °C in buffer containing 25 mM potassium phosphate, pH 7.5; 150 mM KCl; 25% (*v*/*v*) glycerol; 1 mM 2-mercaptoethanol; and 1 mM PMSF. Aliquots (2.5 μL) of ligands were injected into a 0.2 mL cell containing the protein solution to achieve a complete binding isotherm. Protein and ligand concentrations were 20 μM and 200 μM, respectively. The heat of dilution was measured by injection of the ligand into the buffer solution or by additional injections of ligand after saturation; the values obtained were subtracted from the heat of reaction to obtain the effective heat of binding. The resulting titration curves were fitted using the MicroCal Origin software, assuming one set of binding sites. Affinity constants (*K*_a_) and enthalpy variations (Δ*H*) were determined, and the entropy variations (Δ*S*) were calculated from the equation: *T*Δ*S* = Δ*H* + *RT*ln*K*_a_.

### 4.5. Electrophoresis of mOAZ1-Polyamine Complex

The recombinant mOAZ1 protein (1 μg/band) was incubated with 200 μM of a polyamine at room temperature for 30 min. Then the sample was quenched with a loading buffer (200 mM Tris-HCl, pH 6.8, 0.01% bromophenol blue, and 40% glycerol) and applied onto 12% polyacrylamide gel lacking SDS. Electrophoresis was carried out in a running buffer with decreased SDS concentration (25 mM Tris base; 250 mM glycine; and 0.01% SDS) at 70 V. The gel was stained with Coomassie R-250.

### 4.6. Cell Culture

The DU145 cell line was obtained from American Type Culture Collection, Manassas, VA, USA. OAZ1-overexpressing DU145 cells were generated as described earlier [60]. The cells were cultured in humidified atmosphere at +37 °C, 10% CO_2_ in high-glucose Dulbecco’s Modified Eagle’s Medium supplemented with 10% heat-inactivated fetal bovine serum, 2 mM *L*-glutamine, and 50 μg/mL gentamycin. The cells were lysed in a buffer containing 50 mM potassium phosphate, pH 7.2, 0.1 mM EDTA, 0.1% Triton X-100, 1 mM dithiothreitol (DTT), and Complete EDTA-free protease inhibitor (Roche Diagnostics, Basel, Switzerland). Aliquots of the lysate were taken for polyamine measurement, and the rest was centrifuged at 13,000 rpm for 20 min at +4 °C. The supernatant was used for enzyme assays and Western blotting.

### 4.7. [^14^C]-Put and -Spd Uptake Experiments

OAZ1-overexpressing DU145 cells were plated onto 6-well plates and incubated overnight. The cells were first treated with DFMO (5 mM) for 3 days, after which DOX (1 μg/mL) was added for further 2 days to induce OAZ1 overexpression. Then, AG (1 mM), APCHA (50 μM), and Spd/analogue (100 μM) were added for 4 h. The plates were washed with warm DMEM and incubated for 20 min in [^14^C]-Put or [^14^C]-Spd labeling mix (10 μM Put/Spd, specific activity 25 mCi/mmol, in DMEM). The plates were washed twice with ice-cold PBS and lysed to 0.5 mL of 0.1 M NaOH. An aliquot was taken for measurement of ^14^C radioactivity, and the counts were normalized to total protein in the sample.

### 4.8. OAZ1 Half-Life in DU145 Cells

OAZ1-overexpressing DU145 cells were plated onto 100 mm plates and incubated overnight. The cells were treated with DOX (1 μg/mL) for 24 h to induce OAZ1 expression, and then 100 μM polyamine/analogue was added for further 4 h. The cells were incubated with CHX (100 μg/mL) for 0 or 40 min, and the amount of OAZ1 protein was analyzed by immunoblotting.

### 4.9. In Vitro Effect of Spd and Its Analogues on ODC Activity

ODC-enriched lysate was prepared from subconfluent DU145 cells 6 h after medium change by lysing the cells in buffer containing 25 mM Tris-HCl, pH 7.4, 1 mM DTT, and 0.01% Tween-80. OAZ1-enriched lysate was prepared from OAZ1-overexpressing DU145 cells in the same buffer. The lysate was cleared by centrifugation, and the endogenous polyamines were removed by ultrafiltration through 3K-cutoff filter and washing twice with lysis buffer. In experiment 1, ODC-enriched cytoplasmic extract (~100 μg of total protein) was incubated for 30 min at +37 °C in the presence of 1 mM Spd or analogues before ODC activity assay. In experiment 2, ODC-enriched cytoplasmic extract (~100 μg of total protein) was incubated for 30 min at +37 °C with OAZ1-enriched cytoplasmic extract (~100 μg of total protein) in the presence of 1 mM Spd or analogues before ODC activity assay.

### 4.10. RNA Melting Point Experiments

UV thermal denaturation data were obtained on a Hitachi U-2900 spectrophotometer equipped with a Peltier temperature controller. The concentration of model 72-mer-2′-O-Me-oligoribonucleotide L-OM, containing the +1 frameshift site, hairpin, and pseudoknot of the mOAZ1 mRNA was 1 µM. Samples were dissolved in 50 mM Tris-HCl, pH 7.5 and 50 mM NaCl buffer. Concentration of oligonucleotide was determined after quantitating the samples by UV absorbance at λ = 260 nm. Samples were heated to 90 °C for 10 min then cooled slowly to room temperature and stored at 5 °C for at least 18 h before the measurements were performed. Denaturation curves were acquired at 260 nm for the duplexes at a rate of 0.5 °C/min, within a range of 30–90 °C. The *T*_m_ values were determined from polynomial fitting of the observed curves and taken as the temperatures corresponding to half-dissociation of complex [61]. The first derivative of absorption with respect to temperature, dA/dT, of the melting curve was computer-generated by the GraphPad Prism 7.0 software and used for the determining of *T*_m_.

### 4.11. Determination of the Efficiency of Ribosomal Frameshifting Using β-Galactosidase and Firefly Luciferase Reporter System

For this purpose, the earlier developed frameshift reporter system [28], which includes quadruple *S. cerevisiae* mutant strain IS532/44d, unable to synthesize and degrade polyamines due to deletion of genes *FMS1*, *SPE1*, *SPE2*, and *PAA1*, and the plasmids pAC98T-OAZ1-FS and pAC98T-OAZ1-Cont were used. Each of these plasmids bears the reporter gene encoding β-galactosidase and firefly luciferase gene fused with the fragment of eukaryotic *OAZ1* containing frameshift site. The plasmid pAC98T-OAZ1-FS contains native eukaryotic *OAZ1* fragment, so the efficiency of luciferase synthesis and enzymatic activity depend on the +1 frameshift event. The plasmid pAC98T-OAZ1-Cont contains the eukaryotic *OAZ1* fragment with TAG stop codon replaced by TGG tryptophan codon that enables constitutive synthesis of both β-galactosidase and luciferase whose activity is taken as 100%. Bicistronic assays for frameshifting were performed as described earlier [28] with the following modifications. Yeast transformants of IS532/44d strain were grown on agar plates with the synthetic media containing 0.67% (*w*/*v*) Yeast Nitrogen Base without amino acids (Sigma), 2% (*w*/*v*) glucose, 2% (*w*/*v*) agar, mixture of 19 amino acids without leucine (2 mg/mL each), and adenine (2 mg/mL) at 30 °C for 5–7 days. Then, colonies of yeast transformants were grown in the synthetic media lacking leucine and polyamines for two days. Yeast cultures were diluted to OD600 = 0.1 in 5 mL of synthetic media lacking leucine and supplemented with either Spd, 1-MeSpd, 2-MeSpd, or 3-MeSpd, each at final concentrations of 0.25, 0.5, 1, 2, and 4 mM. Yeast cultures were grown to log phase (OD600 = 0.8–1.4) at 30 °C and harvested by centrifugation, and cell pellets were washed three times with water. Cell pellets were resuspended in 500 μL of buffer containing 60 mM NaH_2_PO_4_, 40 mM Na_2_HPO_4_, 10 mM KCl, 1 mM MgSO_4_, 50 mM 2-mercaptoethanol, and 10% glycerol and lysed by vortexing with acid-washed glass beads (Sigma). The lysates were centrifuged (15,000× *g*, 10 min, 4 °C), and the supernatants were used for β-galactosidase and firefly luciferase reporter gene assays. β-Galactosidase activity was analyzed using ο-nitrophenyl-β-D-galactopyranoside (Sigma) as the substrate in accordance with published protocol [62]. Activity of firefly luciferase was analyzed in 96-well plates using Luciferase Assay System (Promega). Frameshift frequencies were calculated as described [28].

### 4.12. Analytical Methods

Polyamines were measured with HPLC according to the published method [63]. Acid-precipitated pellets were dissolved to 0.1 M NaOH, and the amount of DNA was measured using the PicoGreen reagent (Invitrogen) according to manufacturer’s instructions using dilutions of calf thymus DNA (Sigma-Aldrich) as standard curve. Protein concentrations were measured using Bio-Rad protein kit with dilutions of bovine serum albumin (Bio-Rad, Hercules, CA, USA) as standards. ODC activities were measured from the cytosolic fractions as described earlier [64]. For immunoblotting of OAZ1, 20–50 μg of total protein was separated on a 15% SDS-polyacrylamide gel, transferred to a membrane (Immobilon FL, Millipore, Burlington, MA, USA), and immunoblotted with rabbit anti-OAZ1 antibody (a kind gift from Prof. Olli Jänne, University of Helsinki, Finland) and rabbit antiactin (Santa Cruz Biotechnology, cat no sc-1616, used at 1:1000 dilution in 5% nonfat dry milk/PBS-Tween). The secondary antibody used was goat antirabbit (Santa Cruz Biotechnology, Dallas, TX, USA, cat no sc-2004, used at 1:10,000 dilution).

### 4.13. Statistical Analysis

One-way analysis of variance with Tukey’s post hoc test was used for multiple comparisons with the aid of the GraphPad Prism 5.03 (GraphPad Software Inc., San Diego, CA, USA).

## Figures and Tables

**Table 1:** Thermodynamic parameters of the polyamines binding to mOAZ1 as determined by isothermal titration calorimetry ^a^.

Polyamine	Stoichiometry mOAZ1/Polyamine	*K*_a_ ^b^, M^−1^	Δ*H* ^d^, kcal/mole	*T*Δ*S* ^e^, kcal/mole	*K*_d_ ^c^, µM
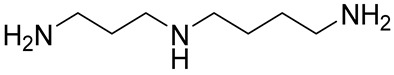 Spd	2:1	2.8 × 10^5^	−3.3	4.2	3.6
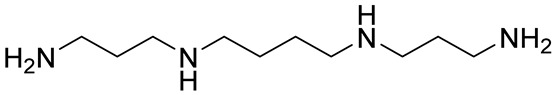 Spm	2:1	9.2 × 10^5^	−3.7	4.5	1.1
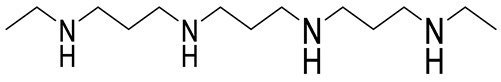 DENSpm	2:1	7.5 × 10^5^	−5.1	3.0	1.3
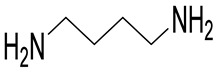 Put	No binding ^f^				
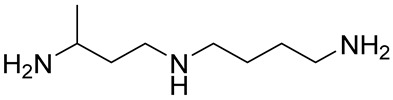 1-MeSpd	2:1	5.4 × 10^5^	−2.2	5.8	1.9
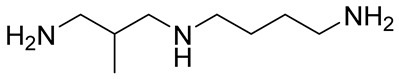 2-MeSpd	No binding ^f^				
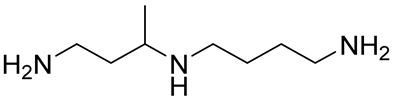 3-MeSpd	2:1	4.0 × 10^5^	−1.2	6.6	2.5

^a^ All the measurements were performed three times in buffer containing 25 mM potassium phosphate, pH 7.5, 150 mM KCl, 25% (*v*/*v*) glycerol, 1 mM 2-mercaptoethanol, and 1 mM PMSF. ^b^ *K*_a_—affinity constant; standard deviation did not exceed ±25%. ^c^ *K*_d_—dissociation constant; calculated as 1/*K*_a_ from experimentally determined *K*_a_. ^d^ Δ*H*—enthalpy variation; standard deviation did not exceed ±20%. ^e^ *T*Δ*S*—entropy variation; calculated from the equation *T*Δ*S* = Δ*H* + *RT*ln*K*_a_. ^f^ No binding means that interaction was not detected in experimental conditions. The concentrations used did not allow to detect *K*_d_ > 50 μM.

## Data Availability

All data are available in the main text or the Supplementary Materials.

## Supplementary Materials

The following supporting information can be downloaded at: https://www.mdpi.com/article/10.3390/ijms23094614/s1.
