# Supplementary material for: Role of Polyamine-Induced Dimerization of Antizyme in Its Cellular Functions
Source: Int J Mol Sci. 2022 Apr 21;23(9):4614. doi: 10.3390/ijms23094614 (PMC9104013; doi:10.3390/ijms23094614)
Supplement: Supplementary file 1 [file ijms-23-04614-s001.zip › ijms-1633104-supplementary.pdf]

Supplementary Material

Role of polyamine-induced dimerization of antizyme in its cellular functions

Mervi T. Hyvönen[1#], Olga A. Smirnova[2#], Vladimir A. Mitkevich[2#], Vera L. Tunitskaya[2], Maxim Khomutov[2], Dmitry S. Karpov[2], Sergey P. Korolev[3], Merja R. Häkkinen[1], Marko Pietilä[4], Marina B. Gottikh[3], Jouko Vepsäläinen[1], Leena Alhonen[1], Alexander A. Makarov[2], Sergey N. Kochetkov[2], Heather M. Wallace[5], Tuomo A. Keinänen[1], Alex R. Khomutov[2]

[1]School of Pharmacy, Biocenter Kuopio, University of Eastern Finland, Kuopio campus, Yliopistonranta 1B, 70210 Kuopio, Finland;
[2]Engelhardt Institute of Molecular Biology, Russian Academy of Sciences, Vavilov Street 32, Moscow, 119991, Russia;
[3]Belozersky Institute of Physico-Chemical Biology, Lomonosov Moscow State University, Leninskie Gory, Moscow, 119991, Russia;
[4]School of Medicine, Biocenter Kuopio, University of Eastern Finland, Kuopio campus, Yliopistonranta 1E, 70210 Kuopio, Finland;
[5]Institute of Medical Sciences, School of Medicine, Medical Sciences and Nutrition, University of Aberdeen, Polwarth Building, Foresterhill, Aberdeen AB25 2ZD, UK.

#Equal contribution

Correspondence to:
mervi.hyvonen@uef.fi
alexkhom@list.ru

This PDF file includes:

Figs. S1 to S5
Tables S1 to S2

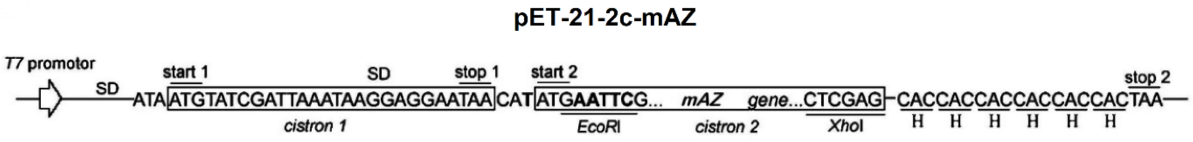

Figure S1. Schematic representation of OAZ1 expression vector pET-21-2c-mAZ.

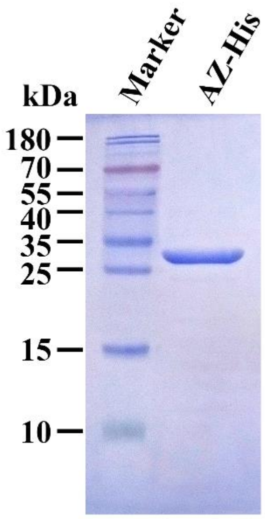

Figure S2. The purified *C*-terminally His-tagged mOAZ1 protein used in this study. mOAZ1-6xHis (1 µg/band) was resolved by SDS-PAGE by using a 12% gel. The higher molecular mass of the recombinant protein than that of the authentic 29 kDa OAZ1 protein is explained by the six His residues and additional *N*-terminal residues encoded by the expression vector.

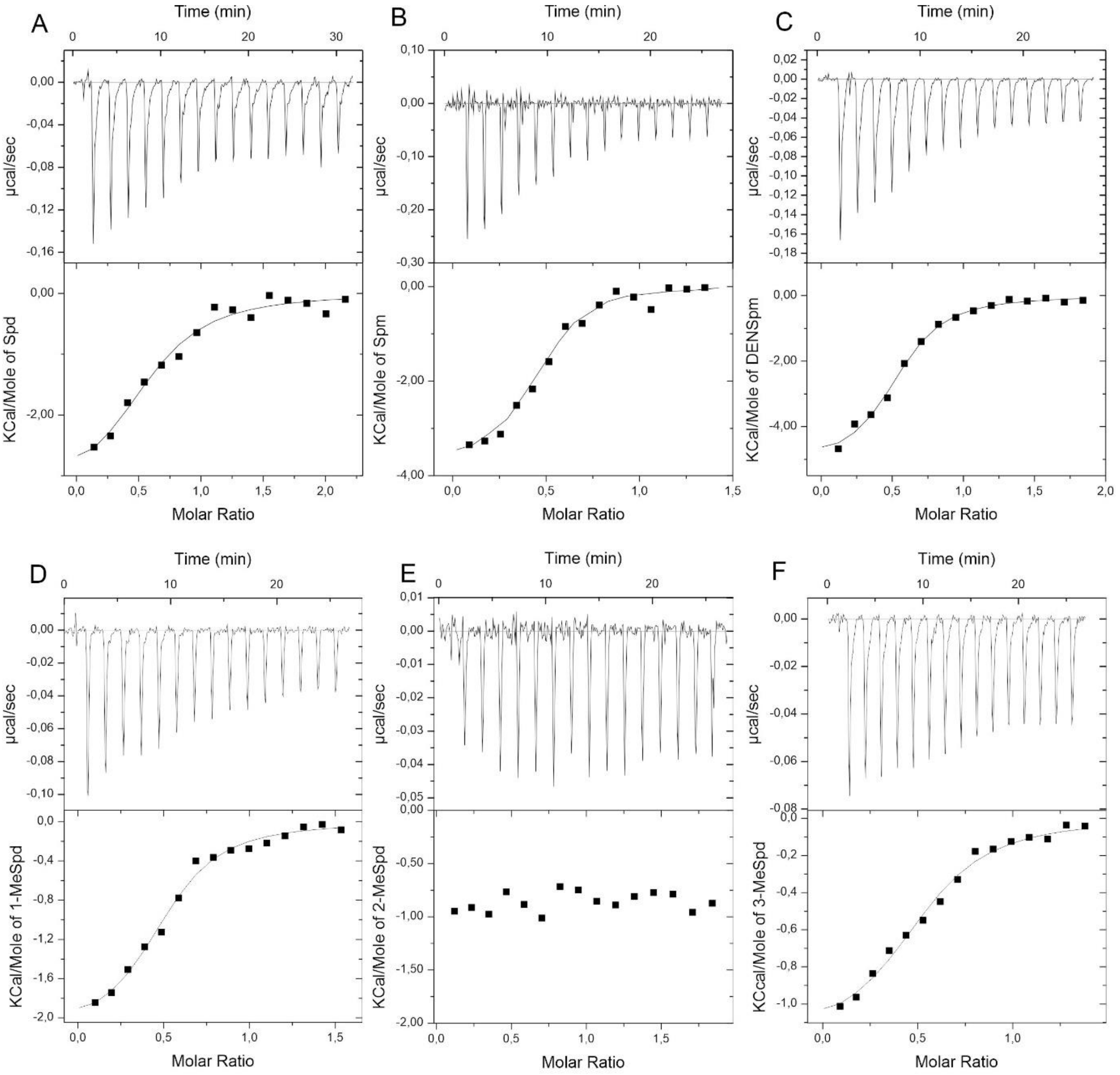

Figure S3. mOAZ1 interaction with polyamines measured by ITC. Titration curves (upper panels) and binding isotherms (lower panels) for mOAZ1 (20 µM) binding to (A) Spd, (B) Spm, (C) DENSpm, (D) 1-MeSpd, (E) 2-MeSpd, and (F) 3-MeSpd (200 µM each) at 31°C, pH 7.5.

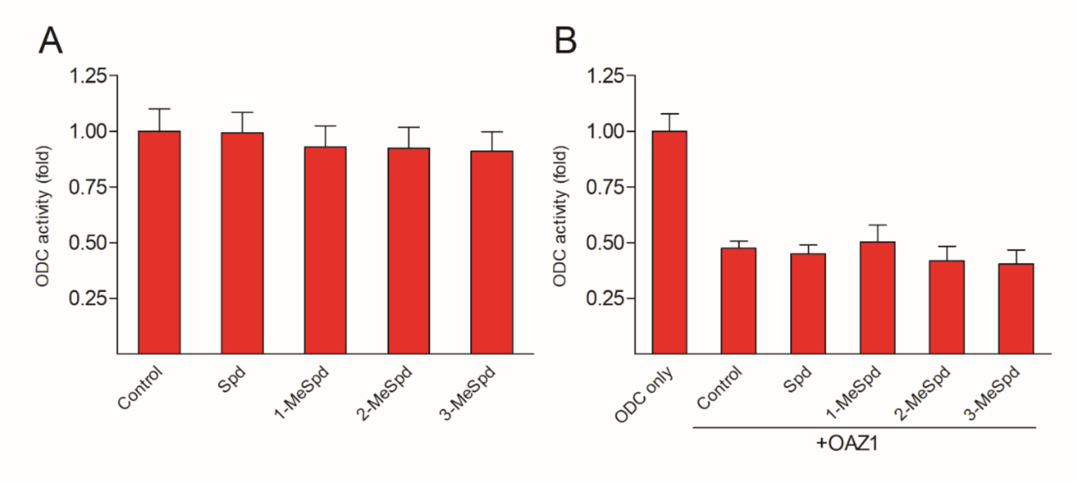

Figure S4. Effect of direct addition of Spd or its analogues (1 mM) on ODC activity in DU145 cytosolic extract. (A) ODC-enriched cytosolic extract was incubated for 30 min at +37°C with Spd or its analogues, and ODC activity was then assayed. (B) ODC-enriched cytosolic extract (ODC only) was incubated for 30 min at +37°C with cytosolic extract from OAZ1-overexpressing cells and with Spd or its analogues, and ODC activity was then assayed. Results are means ± SD, n=3.

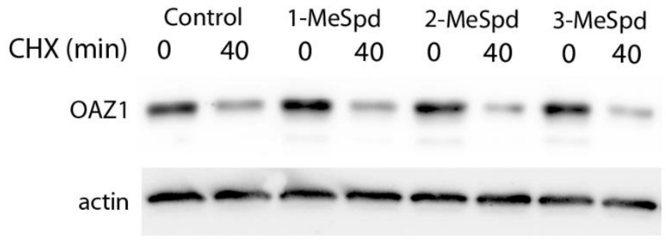

Figure S5. Degradation of OAZ1 in DU145 cells is not affected by *C*-methylated Spd analogues. OAZ1 was induced by overnight incubation with DOX (1 μg/ml), after which analogues (100 μM) were added for further 4h. Then, CHX (100 μg/ml) was added, and samples collected after 40 min for Western blotting analysis.

Table S1. Effect of Spd and its *C*-methylated analogues on the melting temperature (T_m) of 72- mer -2'-O-Me-oligoribonucleotide L-OM, containing the +1 frameshift site, hairpin and pseudoknot of the mOAZ1 mRNA (1 μM). Results are means ± SD, n=3. *, ** and *** refer to statistical significance of $p<0.05$, $p<0.01$ and $p<0.001$, respectively, as compared to Spd group of same concentration.

Polyamine concentration, μM	T_m, °C			
	Spd	1-MeSpd	2-MeSpd	3-MeSpd
0	65.5±0.6			
20	73.7±0.6	73.0±0.4	70.5±0.5***	71.9±0.5*
50	75.3±0.4	74.0±0.3	73.4±0.4*	75.2±0.4
200	79.7±1.1	80.2±0.6	77.5±0.5**	79.4±0.5

[a]T_m measurements were performed in at least three separate experiments, with 1 μM **L-OM:** 72-chain-2'-O-Me-oligonucleotide (5'-UGG UGC **UCC UGA** UGU CCC UCA CCC ACC CCU GAA GAU CCC AGG UGG GCG AGG GAA CAG UCA GCG GGA UCA CAG-3') in 50 mM Tris-HCl pH 7.5, 50 mM NaCl buffer.

Table S2. Intracellular polyamine and analogue concentrations in DU145 cells after 4 h incubation with *C*-methylated Spd analogues (each 100 µM) in the presence of 1 mM AG. Results are means ± SD, n=3.

	Put	Spd	Spm	N^1-AcSpd	Analogue	Spd + MeSpd	Spd + Spm + MeSpd	Total polyamines
	(pmol/µg DNA)							
Control	31.0 ± 2.7	135 ± 7.6	81.2 ± 3.4	4.8 ± 0.5		216	216	252
1-MeSpd	9.5 ± 0.5	62.0 ± 3.9	74.0 ± 7.2	7.1 ± 0.2	170 ± 9.7	232	306	323
2-MeSpd	21.1 ± 0.7	70.2 ± 2.3	83.5 ± 6.8	4.9 ± 0.3	126 ± 14.5	196	279	306
3-MeSpd	11.1 ± 0.2	65.3 ± 3.9	78.4 ± 5.8	5.9 ± 1.1	103 ± 1.9	168	246	264
